# Progression of herpesvirus infection is inhibited by calcium reporter

**DOI:** 10.17912/micropub.biology.001269

**Published:** 2024-08-19

**Authors:** Kari Kunnas, Maija Vihinen-Ranta, Simon Leclerc

**Affiliations:** 1 Department of Biological and Environmental Science and Nanoscience Center, University of Jyväskylä, Jyvaskyla, Central Finland, Finland

## Abstract

During infection, Herpes simplex virus type 1 (HSV-1) alters the mitochondrial structure and function of the host cell. Live-cell imaging with fluorescent reporters revealed increased mitochondrial calcium and a transient ROS enrichment after HSV-1 infection. Notably, cells co-transfected with a calcium reporter displayed smaller viral replication compartments, while those with a ROS reporter exhibited average growth of viral replication compartments. Our findings suggest that the virus-induced increase in mitochondrial calcium, followed by an increased amount of bound calcium reporter, interferes with the progression of the infection.

**Figure 1. HSV-1 infection increases mitochondrial Ca²⁺ and ROS:**
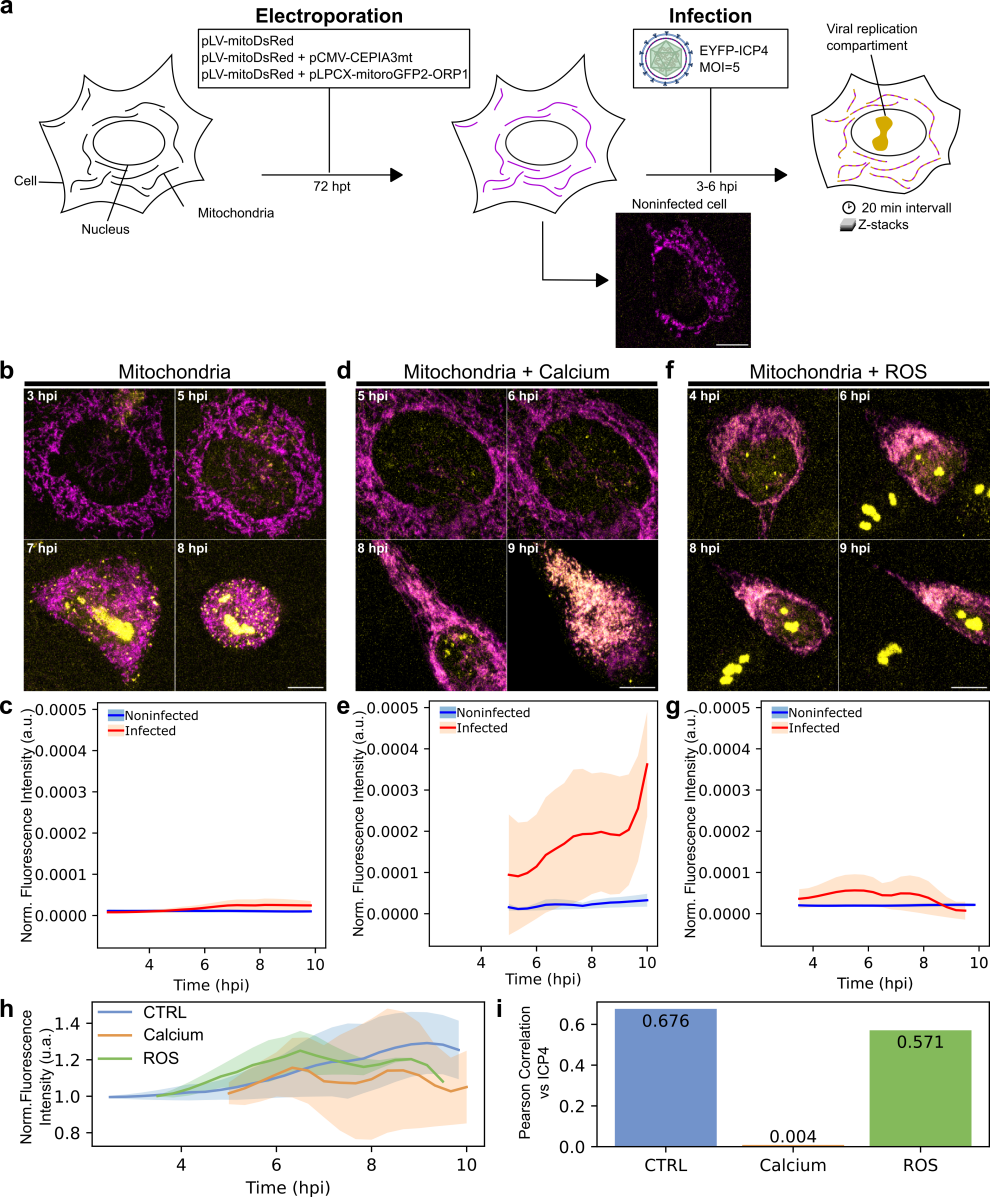
( **a** ) Schematic protocol, where mouse embryonic fibroblast (MEF) cells are transfected with one or two plasmids encoding mitochondria-targeted fluorescent proteins, and 72 h later, infected by HSV-1 (yellow). A noninfected transfected (DsRed) representative cell is shown. After 3 to 6 hpi, a cell z-stack is imaged every 20 min. ( **b** ) Cellular distribution of mitochondria (magenta), ( **d** ) mitochondrial calcium (yellow/pink), and ( **f** ) mitochondrial ROS (yellow/pink) are shown in infected cells at different time points of the infection. Viral EYFP-ICP4 shows the presence of viral replication compartments (yellow). Scale bars, 5 μm. The normalized fluorescence intensity measurements of ( **c** ) mitochondria, ( **e** ) calcium, and ( **g** ) ROS markers. The continuous line indicates the mean, while the semi-transparent area illustrates the standard deviation (noninfected blue; infected red). ( **h** ). The nuclear fluorescence intensity of EYFP-ICP4 in the presence of mitochondrial, calcium and ROS markers. ( **i** ) Pearson correlation between the amount of viral EYFP-ICP4 and mitochondrial reporters

## Description

Mitochondria, the powerhouses of the cell, are essential for ATP production through oxidative phosphorylation. These complex organelles also play a crucial role in the innate immune response (Sorouri, Chang, and Hancks 2022) , particularly through signalling pathways involving calcium (Ca²⁺) (Giorgi, Marchi, and Pinton 2018) and reactive oxygen species (ROS) (Sena and Chandel 2012) . Mitochondrial Ca²⁺ regulates diverse cellular processes. Elevated mitochondrial Ca²⁺ can enhance the Krebs cycle, increasing ATP production to meet cellular energy demands (Traaseth et al. 2004) . Conversely, excessive Ca²⁺ can cause the opening of the mitochondrial permeability transition pore, triggering cell death pathways (Endlicher et al. 2023) . ROS, generated as by-products of the mitochondrial electron transport chain, are signalling molecules affecting gene expression and cell survival (Miller et al. 2019) . However, elevated levels of ROS disrupt cellular homeostasis, causing oxidative stress and damage to biomolecules (Kowalczyk et al. 2021) .

The balance between Ca²⁺ and ROS within mitochondria is critical in modulating the cellular response to viral infections. However, viral counteractions can manipulate mitochondrial functions, cellular metabolism, and immune responses to facilitate viral replication and spread (Foo et al.,2022). Herpes simplex virus type 1 (HSV-1) is a common human pathogen that can cause various infections, from cold sores to more serious neurological diseases. During infection, HSV-1 reorganizes mitochondrial morphology (Leclerc et al. 2024) and function (Vastag et al. 2011) . Our live-cell studies demonstrate that HSV-1 infection induces a substantial increase in mitochondrial Ca²⁺ and a moderate transient increase of ROS.

To investigate the impact of HSV-1 infection on mitochondrial function in mouse embryonic fibroblast (MEF) cells, we employed live-cell imaging of transiently transfected plasmids expressing fluorescent reporters of mitochondria, Ca²⁺, and ROS. The mitochondria were visualized by fluorescent protein (mitoDsRed) targeted to the mitochondria. Ca²⁺ was detected by a genetically encoded indicator (CMV-CEPIA3mt) and ROS by its indicator (LPCX-mito roGFP-ORP1). Cells were infected 72 hours post-transfection with HSV-1 at a multiplicity of infection (MOI) of 5. Viral replication and localization of a viral replication compartment were verified by the expression of a viral EYFP-ICP4 fusion protein (Fig1. a). The emission spectra of the mitochondrial indicators and EYFP-ICP4 are similar (yellow). However, the nuclear localization of EYFP-ICP4 and the cytoplasmic presence of indicators allowed their separation during analysis.

As previously described (Kobiler et al. 2011; Simpson-Holley et al. 2005; Aho et al. 2017) , our studies confirm the emergence of small, dispersed viral replication compartments at 5 hours post-infection (hpi), followed by their fusion into larger compartments at 7-8 hpi. Later, at 12 hpi, extensive cytopathic effects are observed, including rounded cells with disrupted nuclear membranes, chromatin marginalization, and cell detachment from the coverslip (Fig1. b). This was potentially due to the combined effects of viral infection and extended observation. The viral reporter EYFP-ICP4 expression and the condensation of the mitochondria towards the nuclear envelope followed the progression of infection. In infected cells transfected with mitoDsRed, the viral replication compartment growth and fusion and the change in mitochondria and cell morphology are visible (Fig1. b). The yellow fluorescence of mitochondria remained near zero without mitochondrial function reporter (Fig1. c).

In cells co-transfected with the mitoDsRed and Ca²⁺ reporter, the viral replication compartment started to form at 4 hpi but failed to expand later in infection (Fig1. d). An increased amount of Ca² accompanied the progression of infection with a significant increase between 5 and 10 hpi (Fig1. e). In cells co-transfected with the mitoDsRed and ROS reporter, the viral replication compartment formed was similar to control cells (Fig1. f). Notably, cells started to detach from the coverslip after 6 hpi and the amount of ROS decreased as the infection proceeded (Fig1. g). This suggests that dual transfection of infected cells with a mitoDsRed and ROS reporter led to disturbance of cellular morphology and cell death faster than only dsRed transfected cells. The analysis of the mitochondrial reporters during the progression of infection showed that the intensity of signals of markers fluctuated when the intensity of EYFP-ICP4 and viral replication compartment increased ( Fig. 1 h). In mitoDsRed-labeled control cells, a positive correlation between mitoDsRed and EYFP-ICP4 was observed, reflecting the higher cytoplasmic background resulting from EYFP-ICP4 produced during successful infection. Interestingly, the calcium reporter showed no correlation with EYFP-ICP4, verifying the inhibition of viral replication in these cells. Finally, the ROS reporter displayed a moderate positive correlation with viral protein, suggesting some ROS production even with reduced viral replication ( Fig. 1 i).

Altogether, our results show that the formation of an enlarged viral replication compartment was inhibited in cells transfected with Ca²⁺ reporter. Our previous studies demonstrated an infection-induced increase in the overall cellular Ca²⁺ levels (Leclerc et al. 2024) . Our results suggest that the increased presence of calcium leads to elevated levels of reporters. While the calcium-binding affinity of the calcium reporter might be weak, its interaction with Ca²⁺, specifically within the mitochondria could interfere with the progression of infection. This hypothesis needs further investigation, such as using mitochondrial Ca²⁺ chelators like Ruthenium Red (Marmolejo-Garza et al. 2023) , which could help elucidate whether manipulating the available mitochondrial Ca²⁺ pool influences HSV-1 replication dynamics. It is also crucial to consider other factors, such as transfection methods or plasmid constructs, that might impact cell viability. Therefore, using different calcium reporter variants (Kanemaru et al. 2020) could help confirm our findings.

## Methods

**Cells and viruses**

Mouse embryonic fibroblast cells (MEF, ATCC CRL-2991) were grown in Dulbecco’s modified Eagle medium (DMEM) supplemented with 10% foetal bovine serum, L-glutamine, and penicillin-streptomycin (Gibco-Invitrogen, Carlsbad, CA) at 37°C in the presence of 5% CO2.

**Electroporation**

MEF cells were trypsinized, counted, and 5 x 10 ^5^ cells were resuspended in 110 µl of resuspension buffer (Invitrogen). 4 μg of each plasmid was added to the cell and electroporated (Neon transfection system, Invitrogen) using a single pulse of 30 ms at 1350 volts. After electroporation, cells were split into two wells at a 0.7 to 0.3 ratio. The cells were then grown on a square coverslip in 6 well plates for 72 hours.

**Viral infection**

On the day of infection, cells were infected with the EYFP-ICP4 (vEYFP-ICP4) strain (Everett et al. 2004) at a multiplicity of infection (MOI) of 5. After one hour, the culture medium was replaced with a phenol-free imaging medium (same formulation, Gibco-Invitrogen, Carlsbad, CA) and incubated for 3-5 hours at 37°C with 5% CO2 before imaging.

**Microscopy and image acquisition**

Cells were imaged on a Leica TCS SP8X Falcon confocal microscope (Leica Microsystems, Mannheim, Germany) with a water immersion objective (HC PL APO CS2, NA: 1.2) and a HyD detector. Images were acquired at 3x zoom with a pixel size of 190 nm. Z-stacks consisted of 16 planes spaced 500 nm apart. To minimize photobleaching and crosstalk, YFP/roGFP/CEPIA (excitation: 498 nm, emission: 508-545 nm) and DsRed (excitation: 560 nm, emission: 580-650 nm) were acquired sequentially. Autofocus (Leica Microsystems, Mannheim, Germany) compensated for Z-drift during the acquisition. Each Z-stack acquisition took 30-40 seconds. 20 cells were imaged at 20-minute intervals over 12 hours.

**Image processing and analysis**

Following image acquisition, individual cells were isolated from the acquired Z-stacks. This involved cropping the images to eliminate background and signals from neighbouring cells. To segment mitochondria within each isolated cell, we employed the dsRed-Mito7 channel and the Otsu thresholding method. Next, we quantified two parameters for each cell across the entire timelapse: mitochondrial volume and raw green fluorescence intensity. The mitochondrial volume provides information about the overall size of the mitochondria network, while the raw green fluorescence intensity, measured from the mitochondrial function reporters (CEPIA or roGFP-ORP1), reflects the reporter activity within the mitochondria. To account for potential variations in mitochondrial size, the green fluorescence intensity was normalized by dividing it by the corresponding mitochondrial volume for each cell. We further analysed the normalized green fluorescence by calculating average traces for each condition, followed by smoothing the average trace using a Savitzky-Golay filter (Poly-order 3). A similar procedure was applied to obtain average and smoothed traces for the standard deviation.

Sample size:

- Control (pLV-mitoDsRed only)
  - Noninfected cells: 11
  - Infected cells: 9

- Calcium (pLV-mitoDsRed and pCMV CEPIA3mt)
  - Noninfected cells: 10
  - Infected cells: 6

- ROS (pLV-mitoDsRed and pLPCX mito roGFP2-ORP1)
  - Noninfected cells: 6
  - Infected cells: 5

## Reagents

**Table:** 

**AddGene reference**	**Name**	**Reference**
58219	pCMV CEPIA3mt	(Suzuki et al. 2014)
44386	pLV-mitoDsRed	(Kitay et al. 2013)
64977	pLPCX mito Grx1-roGFP2	(Gutscher et al. 2008)
